# Dataset and multivariate statistical tools for mathematical modeling of water desorption isotherms and infrared spectral properties of cupuassu (*Theobroma grandiflorum* L.) pulp

**DOI:** 10.1016/j.dib.2025.111720

**Published:** 2025-05-28

**Authors:** Andrés F. Bahamón-Monje, Paola A. García-Rincón, Gentil A. Collazos-Escobar, Claudia M. Amorocho-Cruz, Nelson Gutiérrez-Guzmán

**Affiliations:** a Centro Surcolombiano de Investigación en Café (CESURCAFÉ), Departamento de Ingeniería Agrícola, Universidad Surcolombiana, Neiva-Huila 410001, Colombia; b Departamento de Ingeniería Agroindustrial, Facultad de Ingeniería, Universidad Surcolombiana, Neiva, Huila, Colombia; c Grupo de investigación de Biotecnología y Control de Calidad de Alimentos, Universidad de la Amazonia, 180001 Florencia-Caquetá, Colombia; d Grupo de Análisis y Simulación de Procesos Agroalimentarios (ASPA), Instituto Universitario de Ingeniería de Alimentos–FoodUPV, Universitat Politècnica de València, Camí de Vera s/n, Edificio 3F, València 46022, Spain

**Keywords:** Model-based investigation, Gibbs free energy, Low-temperature drying, Energy requirements, Non-spontaneous process, Infrared spectral analysis, Unsupervised machine learning, Quality monitoring

## Abstract

This study presents a comprehensive dataset of water desorption isotherms and infrared spectral data for cupuassu pulp, a by-product with significant potential for value-added applications in the food industry. The dataset includes experimentally determined desorption isotherms within a water activity range of 0.1–1 at 25 °C, obtained using the Dynamic Dewpoint Isotherm (DDI) method. Infrared spectra were acquired via Attenuated Total Reflectance Fourier Transform Infrared (ATR-FTIR) spectroscopy, covering the wavenumber range of 4000–650 cm^–1^ with a resolution of 8 cm^–1^. Additionally, mathematical modeling and multivariate statistical tools were applied to analyze water desorption behavior, optimizing low-temperature energy consumption and ensuring maximum storage stability. Preprocessing techniques, including baseline correction, Standard Normal Variate (SNV), and Multiplicative Scatter Correction (MSC), were employed to enhance spectral data quality. Principal Component Analysis (PCA), based on scores-loadings computation, was utilized for exploratory analysis of the cupuassu pulp spectral fingerprint. The dataset is provided in Excel format, organized by experimental conditions and replicates. Moreover, MATLAB® R2023a (The MathWorks Inc., Natick, MA, USA) scripts for multivariate statistical analysis were implemented to facilitate model-based assessment of water desorption and infrared properties. In this regard, two MATLAB scripts detail the step-by-step mathematical modeling of isotherms using Peleg, Caurie, and White & Eiring sorption models, as well as Gibbs free energy computation for moisture stability optimization. Furthermore, two MATLAB scripts are dedicated to spectral preprocessing and PCA: one prompts the user to select preprocessing techniques, including the option for sequential application, and visualizes the resulting infrared spectra; the other enables PCA calibration based on the selected preprocessed data. This research provides valuable insights for the food industry, supporting informed decision-making in cupuassu pulp processing. By improving food-processing strategies, ensuring product consistency, and optimizing dehydration processes, this dataset contributes to the development of value-added products from cupuassu by-products, promoting sustainability and resource efficiency.

Specifications Table

**Table:** 

Subject	Food Engineering
Specific subject area	Food technology, Food Science
Type of data	Excel files (Water desorption isotherms, raw infrared spectra, baseline corrected infrared spectra, SNV infrared spectra and MSC infrared spectra). MATLAB files (Modeling water desorption isotherms, Gibbs free energy analysis, Peleg, Caurie and White and Eiring model fitting, spectral preprocessing, baseline, SNV and MSC corrections and explorative principal component analysis). Figures (Experimental and predicted water desorption isotherms, Gibbs free energy analysis, independent and sequential preprocessed infrared spectra).
Data collection	Water desorption isotherm data (Dynamic Dewpoint Isotherm (DDI) analysis). Infrared spectra (Attenuated Total Reflectance-Fourier Transform Infrared, ATR-FTIR).
Data source location	The experimental dataset presented in this study was obtained by the Centro Surcolombiano de Investigación en Café (CESURCAFÉ) at Universidad Surcolombiana, Neiva-Huila, Colombia.
Data accessibility	Repository name: Mendeley Data Data identification number: 10.17632/r57xz7mm82.2 Direct URL to data: https://data.mendeley.com/datasets/r57xz7mm82/2
Related research article	None.

## 1 Value of the Data

• This dataset presents comprehensive water desorption isotherms for cupuassu pulp, elucidating the moisture behavior of the pulp at varying relative humidity levels. It offers critical insights into the hygroscopic properties of cupuassu pulp, which are essential for optimizing drying, storage, and processing conditions. These insights are vital for developing strategies aimed at enhancing the value of this food byproduct.
• The integration of infrared spectral data allows for precise characterization of the chemical composition of cupuassu pulp, ensuring the preservation of its functional and bioactive properties, which is crucial for food and pharmaceutical applications.
• The dataset supports the development of predictive mathematical models, facilitating the optimization of drying process, reducing time and energy consumption, and improving industrial efficiency.
• The use of multivariate statistical tools, such as PCA-based chemometric models, enhances data interpretation by identifying patterns and compositional variations among cupuassu pulp samples from different regions, aiding in quality control and the standardization of strategies to valorize this by-product.
• This research provides valuable information for the food industry by enabling better decision-making regarding cupuassu pulp processing, ensuring product consistency, improving storage strategies, and contributing to the development of value-added products from cupuassu by-products.

## 2 Background

Cupuassu (*Theobroma grandiflorum* L.) is a native Amazonian fruit, valued for its unique sensory characteristics, such as aroma, flavor, and texture [1]. Its seeds are used to produce chocolate-like products, while its nutrient-rich pulp is widely used in juices, ice creams, yogurts, desserts, and jams [2]. With a high water content (approximately 84 %) and elevated water activity (a_w_), cupuassu pulp, as well as any high-moisture food product, requires effective drying to ensure stability and prevent microbial spoilage [3]. Thus, understanding water desorption isotherms is crucial for optimizing drying processes, as these isotherms describe the relationship between water content and surround humidity, aiding in the determination of optimal moisture levels [4]. Moreover, thermodynamic parameters such as Gibbs free energy helps to estimate energy requirements for moisture removal [5]. However, comprehensive studies on the desorption behavior of cupuassu pulp are limited, highlighting the need for applied researches. Fourier Transform Infrared spectroscopy offers an efficient method for analyzing the chemical composition of cupuassu pulp, preserving valuable bioactive compounds. Infrared spectral data can be processed using multivariate statistical tools, such as PCA, to identify compositional variations among pulp samples from different regions [6]. Integrating water desorption and infrared spectral data could optimize drying processes [7], improve product quality, and enhance the valorization of cupuassu pulp by-products in the food and pharmaceutical industries.

The scientific novelty of this study lies in its integrated analysis of water desorption isotherms and mid-infrared spectra specifically applied to *Theobroma grandiflorum* (Cupuassu) pulp, a fruit for which detailed desorption behavior and spectroscopic characterization have been scarcely explored. While sorption isotherms and infrared spectroscopy analyses have been widely applied to various food products [4,6,[8], [9], [10]], comprehensive studies focusing on cupuassu pulp are limited despite its commercial importance and high perishability due to elevated moisture content [11]. By simultaneously investigating the pulp’s desorption thermodynamics and infrared fingerprint, this study provides valuable insights to optimize drying strategies, preserve bioactive compounds, and maintain the sensory qualities critical for industrial applications. Furthermore, the combination of this dataset with multivariate statistical models provides an innovative approach to differentiate cupuassu pulp quality based on infrared spectra, thereby contributing to better standardization, valorization, and industrial utilization of this Amazonian resource.

## 3 Data Description

The experimental dataset was summarized into different Excel and MATLAB files according to the initial characterization of cupuassu pulp samples and their water desorption and infrared properties. Therefore, the dataset descriptions are presented separately for characterization, desorption isotherms and infrared spectra.

### 3.1 Initial characterization

#### 3.1.1 Cupuassu_InitialCharacterization.xlsx

This dataset contains detailed physicochemical data collected from cupuassu samples obtained from three different growing areas: Zone 1, Zone 2, and Zone 3 (first column in the Excel file). For each zone, nine replicates (n=9; column 2 in the Excel file) were analyzed to ensure reliable and representative data (Table 1). The dataset includes the following key parameters, which provide essential insights into the composition and characteristics of the pulp samples. Moisture content (percentage in dry basis; % d.b; column 3 in the Excel file) indicates the amount of water present in the sample. Water activity (column 4 in the Excel file) measures the availability of water for microbial growth and chemical reactions. Titratable acidity (citric acid %; column 5 in the Excel file) quantifies the acidity of the sample by measuring the amount of titratable citric acid, expressed as a percentage. pH (column 6 in the Excel file) reflects the cupuassu sample’s overall acidity or alkalinity, with lower values indicating acidic conditions and higher values indicating basic or alkaline conditions. Brix (20 °C; column 7 in the Excel file) measures the soluble solids content, typically sugars, presented in cupuassu pulp at 20 °C. Total lipids (% d.b.; column 8 in the Excel file) indicates the total lipid content in the sample, expressed as a percentage of the dry weight and crude protein (% d.b.; column 9 in the Excel file) measures the total crude protein content in the sample, expressed as a percentage of dry weight.

**Table 1:** Initial characterization of cupuassu samples.

Area	Moisture contents (% d.b.)	Water activity	Titratable activitys (citric acid %)	pH	Brix (20 °C)	Total lipids (% d.b.)	Crude proteins (%)
**Zone 1**	5.604 ± 0.772	0.991 ± 0.003	2.336 ± 0.168	3.709 ± 0.079	12.347 ± 0.171	3.441 ± 0.363	7.647 ± 0.559
**Zone 2**	4.740 ± 0.355	1.000 ± 0.003	2.593 ± 0.127	3.600 ± 0.060	12.409 ± 0.182	5.609 ± 0.902	7.292 ± 1.028
**Zone 3**	5.373 ± 0.443	1.000 ± 0.003	1.970 ± 0.174	3.672 ± 0.032	12.459 ± 0.239	4.431 ± 1.518	7.574 ± 0.500

Results are expressed as mean ± standard deviation of n=9 replicates per each zone.

Measuring key physicochemical parameters in cupuassu pulp, a byproduct with significant potential for use in the food industry, is crucial for understanding its quality, stability, and suitability for processing. The moisture content is a fundamental parameter that directly influences the shelf life, texture, and processing behavior of food materials. Water activity is another vital factor in food preservation. Controlling moisture content and a_w_ in cupuassu pulp is essential for maintaining microbial stability and ensuring its compatibility with food formulations. Proper moisture content helps extend the shelf life and prevent spoilage, while lower water activity limits microbial growth and reduces the likelihood of undesirable chemical reactions, such as enzymatic browning, thereby preserving both the appearance and quality of the pulp during processing. Titratable acidity provides insight into the acidic nature of cupuassu pulp, influencing its flavor, preservation, and interactions with other ingredients. High acidity enhances the pulp’s preservation potential by inhibiting microbial growth and promoting stability. Similarly, the pH level is crucial for understanding the pulp’s chemical reactivity and its suitability for processes like fermentation or preservation, while also affecting its flavor and texture, which is important for products like beverages and jams. The Brix value, which measures the concentration of soluble solids like sugars, is essential for evaluating the pulp’s sweetness, fermentability, and overall processing behavior, ensuring the desired consistency and sweetness in food products. Finally, lipids and protein are crucial components of cupuassu pulp. Lipids significantly impact the pulp’s texture, flavor, and nutritional profile, making it suitable for formulations that require specific fat compositions, such as creams, oils, or energy-dense foods. Protein content, on the other hand, is essential for the nutritional value and functionality of food products. High-protein cupuassu pulp can be used in protein-enriched foods or as a texture enhancer in various food formulations.

Thus, cupuassu pulp requires special research, since it has a high nutritional value and considerable antioxidant activity, mainly due to phenolics and ascorbic acid [1]. The inherent acidity of cupuassu pulp and its elevated pectin concentration are key attributes that facilitate its use in the production of nectars, jellies, and jams [12]. The titratable acidity (percentage of citric acid content), ranging between 2–3 % across the three growing areas analyzed (Table 1), closely aligns with the findings reported by [13] and is slightly lower than the levels documented by [14]. According to [15], unfermented cupuassu beans exhibit a pH of approximately 6.14, contrasting with the naturally acidic pH of fresh cupuassu pulp, which typically ranges around 3.6 [16]. This acidic environment plays a critical role in stabilizing the viscous structure of the pulp through pectin interaction [12], attributed to the presence of organic acids such as citric, malic, oxalic, lactic, and acetic acids [17]. Moreover, understanding the initial pH of the pulp is vital for accurately determining the optimal fermentation time for the beans [17]. The pH values reported in Table 3 were consistent with those observed by [18], who described cupuassu pulp as having strong acidity, typically with pH values around 3.4.

Regarding lipid content, the total lipid in the pulp falls within the range established by [19], indicating that the lipid concentration remains within expected parameters and does not represent a substantial caloric contribution, especially when compared to the seeds, which possess lipid levels approaching 22 % d.b. The soluble solids content (°Brix) measured in the cupuassu pulp closely corresponded to the values reported by [20]. According to the CODEX STAN 247 (2005) standards, the minimum °Brix value required for cupuassu juice and purée is 9.0 %, and 35.0 % for nectars, affirming the pulp's suitability as a high-quality raw material for further agro-industrial applications. In terms of crude protein content (%), the values obtained were comparable to those reported by [21], who found approximately 8.8 % protein in cupuassu pulp from various regions. The results presented in Table 1 demonstrate that cupuassu pulp samples meet these nutritional recommendations, making the fruit an appealing dietary option without contributing to excessive caloric intake.

### 3.2 Water desorption dataset and mathematical modeling

Experimental water desorption isotherms of cupuassu pulp were compiled in the Excel file “**WaterDesorptionIsotherms_Cupuassu.xlsx”**. This dataset contains the water desorption isotherms of cupuassu pulp at a fixed temperature of 25 °C. It consists of 111 sorption data points (rows), each representing a combination of variables (a_w_, temperature and moisture content) that describe the moisture desorption behavior in this byproduct. The first column in the dataset, labelled “Zone”, indicates the different growing areas where the cupuassu samples were obtained. The second column, “Temperature” contains a constant value of 25 °C for all observations, as the study focuses on the desorption behavior of cupuassu pulp at this specific temperature. The third column, “Water activity” represents the a_w_ values, ranging from 0.09 to 1.00 a_w_ (a_w_ points acquired with a resolution of 0.01 a_w_; see EXPERIMENTAL DESIGN, MATERIALS AND METHODS section). The fourth column, “Moisture content (% wet basis)” provides the moisture content of the cupuassu pulp expressed on a wet basis. This value ranges from 8.14 % to 87 %, indicating the proportion of water relative to the total weight of the sample. Additionally, The last column in the experimental dataset expressed the “Moisture content (% dry basis)” as a dry basis, which ranged from 0.089 % to 6.692 %. This representation is useful for comparing moisture levels independently of variations in the total sample mass due to drying or water loss.

**ModelingWaterDesorptionIsotherms.m:** This MATLAB script provides a structured approach to analyzing water desorption isotherms for cupuassu by loading experimental data and fitting it to various mathematical models. Upon execution, the script loads data from an Excel file titled “**WaterDesorptionIsotherms_Cupuassu.xlsx”**. which contains the experimental variables explained above. The user is prompted to choose a fitting model from the following options, user input 1 indicate the use of Peleg model (Eq. 1), user input 2 indicates Caurie model (Eq. 2) and 3 indicates the use of White and Eiring model (Eq. 3; Table 2).

**Table 2:** Mathematical sorption equations used to describe the water desorption isotherms of cupuassu pulp.

Model	Mathematical expression	Reference	Eq.
Peleg	$X_{e}=b_{0}{a_{w}}^{b_{1}}+b_{2}{a_{w}}^{b_{3}}$	[22]	1
Caurie	$X_{e}=\text{exp}\left(b_{1}+b_{2}a_{w}\right)$	[23]	2
White and Eiring	$X_{e}=\;\frac{1}{\left(b_{1}+b_{2}a_{w}\right)}$	[24]	3

where b_i_ (i=0:3) are the model parameters to be optimized using

Based on the user’s selection, the script automatically launches the MATLAB Curve Fitting Tool (cftool) with the appropriate pre-defined fitting file.

**Peleg_Cupuassu.sfit:** This file contains the Peleg model-specific structure and the necessary parameters to fit the data according to the Peleg equation (Eq. 1), which is widely used to describe moisture desorption isotherms in foods. **Caurie_Cupuassu.sfit:** This file contains the Caurie model setup, which is an alternative approach for modeling the desorption isotherms, especially for products with non-linear desorption trend. **White and Eiring_Cupuassu.sfit:** This file contains the structure for fitting data using the White and Eiring model, which is a commonly applied model for describing moisture sorption isotherms in various food materials.

The curve fitting tool of MATLAB software was used to fit experimental water desorption data by finding the model parameters to minimize the difference between the experimental desorption isotherms and those predicted by the model. The statistical procedure performed in Curve Fitting tool included:

**i) Model fitting and parameter estimation:** This step fit the experimental data (a_w_ vs. moisture content) with the selected model. Each sorption equation (Peleg, Caurie, or White and Eiring) are adjusted by the use of Levenberg-Marquardt optimization method combined with the stochastic initialization of these model’s parameters to find the optimal values [25].
**ii) Goodness-of-fit assessment:** This step involves the assessment of predictive performance of adjusted models. This fact is achieved by the computation of several statistical metrics to assess how well the model fits the data. These metrics include the coefficient of determination (R^2^; Eq. 4), adjusted R^2^ (R^2^_adj_; Eq. 5) and the root mean square error (RMSE; Eq. 6). R^2^ and R^2^_adj_ indicate the proportion of variance in the data explained by the model, when higher R² greater predictive performance of model in the mathematical description of water desorption isotherms. While lower values of RMSE value indicates a better goodness fit in the modeling procedure [26].
  (4) $$R^{2}\left(\%\right)=100-\frac{\sum_{i=1}^{N}{\left(X_{\text{exp}}-\;X_{\text{pred}}\right)}^{2}}{\sum_{i=1}^{N}{\left(\bar{X_{\text{exp}}}-\;X_{\text{pred}}\right)}^{2}}$$
  (5) $$R_{\text{adj}}^{2}\left(\%\right)=100-\left(\frac{N-1}{N-M}\right)\left(100-R^{2}\right)$$
  (6) $$\text{RMSE}\;\left(\%d.b.\right)=\sqrt{\frac{1}{N}\sum_{i=1}^{N}{\left(X_{\text{exp}}-X_{\text{pred}}\right)}^{2}}$$
  Where X_exp_ and X_pred_ are the experimental and predicted moisture content, N is the number of experimental sorption data and M is the number of model parameters.
**iii) Residual analysis:** The tool also enables the visualization of residuals, representing the differences between observed and predicted values, to assess the model's fit. This functionality facilitates the identification of potential systematic biases, such as consistent overestimation or underestimation at specific moisture levels.
**iv) Confidence intervals:** The tool computes confidence intervals for the estimated parameters, offering insights into their reliability and precision.
**v) Exporting results:** Once the curve fitting process is complete, the results can be exported for further analysis. The export includes the fitted model parameters, goodness-of-fit statistics (R², R^2^_adj_, RMSE), and the model equation. Additionally, residuals and the fitted curve can be saved for further visualization or reporting. The data can be exported as a **.mat** file, or a script can be generated containing the fitted model equations, enabling reuse in other analyses.
**vi) Model visualization:** The fitted curve is overlaid on the experimental data to visually evaluate how well the model fits. Additionally, plot settings, such as axis labels, legends, and titles, can be customized to improve the clarity and informativeness of the visualization.
**vii) Exporting to MATLAB workspace:** The fitted model and associated data, including parameters and fitting statistics, can be exported to the MATLAB workspace for use in other scripts or analyses. This enables further tasks, such as sensitivity analysis, or the application of the fitted model to additional datasets.

**Fig. 1:**
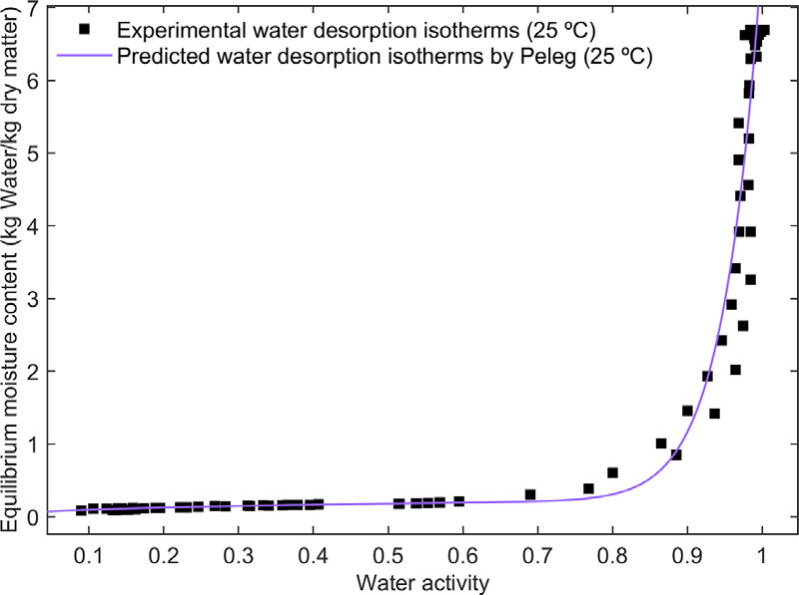
Experimental water desorption isotherms of cupuassu pulp and the predicted fits of the Peleg model.

The statistical results in the model fitting of water desorption isotherms are presented in Table 3. Further, the experimental water desorption isotherms of cupuassu pulp and those predicted by the Peleg fitted model are illustrated Fig. 1.

**Table 3:** Statistical model fitting results for water desorption isotherms in cupuassu pulp.

Product	Model	Parameters-CI (95%)	Goodness of fit
Cupuassu pulp	Peleg	a_0_= 7.53 [7.34, 7.73] a_1_= 19.76 [18.52, 21.04] a_2_= 0.24 [0.07, 0.40] a_3_= 0.39 [–0.21, 0.99]	R^2^= 99.34 % R^2^_adj_= 99.33 % RMSE= 0.21 % d.b.
	Caurie	a_1_= –16.03 [–18.47, –13.58] a_2_= 18.05 [15.55, 20.53]	R^2^_j_= 94.58 % R^2^_adj_= 94.53 % RMSE= 0.61 % d.b.
	White and Eiring	a_1_= 6.38 [6.22, 6.54] a_2_= –6.29 [–6.45, –6.13]	R^2^_j_= 99.38 % R^2^_adj_= 99.37 % RMSE= 0.21 % d.b.

The statistical results in the description of water desorption in cupuassu pulp revealed R^2^ and R^2^_adj_ values ranging between 94.58 % to 99.38 % and RMSE between 0.21 to 0.61 % d.b., indicating high goodness of fit for the calibrated Peleg, Caurie and White and Eiring models in describing the influence of a_w_ on the moisture content for cupuassu pulp. Fig. 1 illustrates the experimental and modeled desorption isotherms of pulp samples. A typical sigmoidal sorption isotherm curve, characteristic of food materials with high sugar and fiber content was observed [27]. At low water activity (a_w_<0.5), the moisture content remains low due to strong water-binding forces, limiting water availability for chemical and microbiological changes. As the water activity increases (a_w_ > 0.75), the moisture content approaches saturation, increasing the risk of microbial spoilage and physicochemical degradation [28].

Based on the water desorption isotherms of cupuassu pulp, the Gibbs free energy can be computed. Gibbs free energy is a reliable thermodynamic property which describes the energy required to make sorption sites available for water molecule attachment [8]. The Gibbs free energy of cupuassu pulp samples was computed using Eq. (7) and the statistical procedure to achieve this aim was compiled in the “**GibbsFreeEnergyAnalysis.m”** MATLAB script.

(7) $$\text{Gibbs}\;\text{free}\;\text{energy}=\text{RT}\;\text{ln}\;\left(a_{w}\right)$$

where R is the universal gas constant for water vapor (8.314 × 10^–3^ kJ mol⁻¹ K⁻¹), and T is the temperature in Kelvin.

In this file “**GibbsFreeEnergyAnalysis.m”** a mathematical data Gibbs free energy-driven model was developed to describe the Gibbs free energy changes as a function of a_w_ and moisture content. This strategy was applied to calibrate a robust model to predict the energy required for drying cupuassu pulp at different a_w_ and moisture levels and to optimizing low-temperature energy consumption ensuring maximum storage stability of cupuassu pulp.

To model the relationship between Gibbs free energy and a_w_, a polynomial model (Eq. 8) was implemented. The script **GibbsFreeEnergyAnalysis.m** prompts the user to select the degree of the polynomial expression for modeling. Furthermore, the file also describes the Gibbs free energy as a function of moisture content (X_exp_) using a double exponential model (Eq. 8).

(8) $$\text{Gibbs}\;\text{free}\;\text{energy}=b_{0}+b_{1}\left(a_{w}\right)+b_{2}{\left(a_{w}\right)}^{2}+\ldots b_{i}{\left(a_{w}\right)}^{n}$$

(9) $$\text{Gibbs}\;\text{free}\;\text{energy}=b_{0}\exp b_{1}\left(X_{\text{exp}}\;\right)+b_{2}\exp b_{3}\left(X_{\text{exp}}\right)$$

Where b_i_ are the empirical model parameters and n is the degree of polynomial expression for modeling Gibbs free energy vs a_w_.

**Fig. 2:**
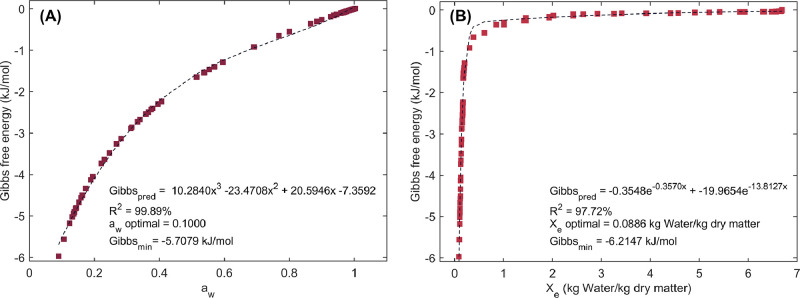
Experimental and predicted Gibbs free energy values of cupuassu pulp. A data-driven mathematical equations were applied to model the Gibbs free energy as a function of water activity (a_w_; A) and moisture content (X_exp_; B).

The adjusted mathematical models (Fig. 2), along with the “fmincon” MATLAB function, were employed to determine the a_w_ and the moisture content that minimize the Gibbs free energy, thereby maximizing the hygroscopic stability of cupuassu pulp. By minimizing Gibbs free energy, the most stable conditions for water retention in the pulp were identified, ensuring optimal preservation during storage. Additionally, these models were used to estimate the energy required for drying the cupuassu pulp at low temperature (Fig. 2), providing valuable insights into the energy efficiency of the drying process while maintaining product quality.

Understanding the desorption isotherms is crucial for optimizing drying and storage conditions for cupuassu pulp. The dataset and multivariate statistical tools could be used to predict drying process, evaluate the product’s shelf life under different humidity conditions, and design drying processes that preserve product quality. Additionally, calculating Gibbs free energy is important to assess the thermodynamic stability of the product and understand the energy changes associated with moisture sorption. This information is especially valuable for the food industry in the formulation and preservation of cupuassu-based products, ensuring their stability, safety, and quality throughout storage and distribution.

The DDI method (Fig. 1) generated a substantial amount of experimental desorption data, which was crucial for gaining a detailed understanding of changes in food matrix interactions during the desorption process. Similar DDI trends have been reported in green and roasted coffee beans [28,29] and pre-mixed powders [30]. The characteristic sigmoid (J-shaped) desorption curve observed in water desorption isotherms of cupuassu pulp (Fig. 1) was consistent with the food’s composition, particularly its richness in soluble components such as sugars and quite similar to agricultural products such as cocoa [31]. Furthermore, similar trends have also been observed in other agricultural products, such as spray-dried fat-filled pea protein powders [32], whole black peppercorns [33] and mango by-products [34]. The Gibbs free energy results (Fig. 2) were consistent with previous findings for curcuma [35] and bean grains [36]. The fitting of the data to data-driven models (Fig. 2A and B) demonstrated a strong correlation and a good fit, allowing the estimation of dehydration limits and energy requirements for the drying process as functions of X_e_ and a_w_.

### 3.3 Infrared spectral data, preprocessing and latent-based mathematical modeling

**RawSpectra_Cupuassu.xlsx:** This Excel file contains infrared spectral data for dried cupuassu samples, acquired using the ATR-FTIR spectroscopy technique (as outlined in the EXPERIMENTAL DESIGN, MATERIALS, AND METHODS section). The dataset is organized as follows: the first column represents the wavenumbers (cm^–1^) across the full infrared spectrum. Columns 2 to 28 contain the spectral data for 27 cupuassu samples collected from three different growing zones. These growing zones represent different cultivation areas and are used to investigate the effect of the cultivation zone on the spectral characteristics of the samples. The columns are grouped into three zones: Zone 1 is represented by columns 2 to 10, containing nine replicates from the first growing zone; Zone 2 is represented by columns 11 to 19, containing nine replicates from the second growing zone; and Zone 3 is represented by columns 20 to 28, containing nine replicates from the third growing zone. Each zone corresponds to a specific region of the spectrum, allowing for detailed analysis and comparison of spectral features across different samples and growing areas. The dataset structure facilitates the examination of spectral variations and the comparison of the effects of different growing areas on the cupuassu infrared spectral properties.

**SpectralPreprocessingCupuassu.m:** The following MATLAB code performs spectral data preprocessing and applies various correction techniques such as baseline correction, SNV normalization, and MSC based on user inputs. The code also visualizes the results of these preprocessing steps through a series of plots. Different steps are presented in the code to

**i) Loading the dataset:** The user is prompted to enter the filename of the spectral data (for this example the dataset name: **RawSpectra_Cupuassu.xlsx**). The data is read into a table and then converted into an array. The first column is assigned to W (representing wavenumbers), and the remaining columns are stored in X matrix (representing the spectral data). The dimensions of the dataset (number of wavenumbers and spectra) are displayed in the command window.
**ii) Baseline correction application:** The user is asked whether to apply baseline correction. If the user chooses to apply it, the polynomial order for the baseline correction is requested. For each spectrum in X matrix, the **BaselineCorrection.m** function is applied using the specified polynomial order. The results are stored in the *SpectraBaseline* matrix.
**iii) SNV application:** The user is asked whether to apply SNV normalization and whether to apply it on baseline-corrected spectra or raw spectra. If SNV normalization is applied to baseline-corrected spectra, the **SNVSpectral.m** function is applied to each column of the *SpectraBaseline* matrix. If applied to raw spectra (**RawSpectra_Cupuassu.xlsx**), the function is applied to the matrix X. The resulting SNV-normalized data is stored in the *SpectraSNV* matrix.
**iv) MSC application:** The user is asked whether to apply MSC and on which dataset (raw spectra, baseline-corrected spectra, or SNV-normalized spectra). If MSC is applied to the SNV-normalized spectra, the mean of SNV is used as the reference spectrum for correction. Similarly, if applied to baseline-corrected or raw spectra, the corresponding mean spectra (*SpectraBaseline* or X matrix) are used as the reference. The **MSCSpectral.m** function is applied to each spectrum in the selected dataset, and the corrected spectra are stored in the *SpectraMSC* matrix.

**BaselineCorrection.m:** This function removes the baseline from a set of spectral data using polynomial fitting. The function takes as input a matrix X (“**RawSpectra_Cupuassu.xlsx**”), where each column represents a spectrum and each row corresponds to a wavenumber, along with a parameter n, which specifies the polynomial degree used for baseline estimation. The function first determines the number of wavenumbers and spectra by examining the dimensions of X and initializes a matrix (*SpectraBaseline*), of the same size as X to store the corrected spectra. The baseline correction is performed by looping through each spectrum (each column of X), fitting an n-degree polynomial to the data using “polyfit” MATLAB function, with wavenumbers as the x-axis. The polynomial is then evaluated using “polyval” MATLAB function, which provides an estimated baseline. This baseline is subtracted from the original spectrum to produce the corrected spectrum. The function outputs *SpectraBaseline*, a matrix containing all spectra with the baseline removed. This method is widely used in spectroscopy to eliminate systematic drifts and background noise, allowing for a more accurate representation of the spectral features of interest [37].

**SNVSpectral.m:** This function applies the SNV transformation to spectral data, a preprocessing method widely used in spectroscopy to correct scattering effects and enhance data consistency [38]. The function receives as input a matrix X (raw or baseline-corrected spectra), where each column represents an individual spectrum, and each row corresponds to a specific wavenumber. It first determines the number of spectra based on the dimensions of X and initializes a matrix of the same size (*SpectraSNV*), to store the transformed spectra. The SNV transformation is performed by iterating through each spectrum, computing the mean spectral value, calculating the standard deviation, and standardizing the data by subtracting the mean and dividing by the standard deviation. This process ensures that each spectrum has a mean of zero and a variance of one, effectively minimizing variations caused by sample scattering. The function outputs *SpectraSNV*, a matrix containing all SNV-corrected spectra, which enhances spectral analysis by normalizing variations associated with differences in sample thickness or particle size, making it an essential preprocessing step for techniques such as chemometric modeling.

**MSCSpectral.m:** This function applies MSC to spectral data using a reference spectrum, a preprocessing technique widely employed in spectroscopy to correct additive and multiplicative scattering effects, thereby improving spectral consistency [38]. This function use as input a matrix X (raw, baseline-corrected or SNV spectra), where each column represents an individual spectrum and each row corresponds to a wavenumber, along with a reference spectrum represented as a column vector, typically the mean spectrum of the dataset. It first determines the number of spectra based on the dimensions of X and initializes a matrix of the same size, *SpectraMSC*, to store the corrected spectra. The MSC correction is performed by iterating through each spectrum, conducting a linear regression between the spectrum and the reference using “polyfit” MATLAB function, which fits a straight line where the intercept represents an additive offset and the slope represents a multiplicative scaling factor. The corrected spectrum is then obtained by adjusting the original spectrum using these regression parameters, effectively removing both additive and multiplicative effects. The function outputs *SpectraMSC*, a matrix containing the MSC-corrected spectra, which is particularly beneficial in minimizing spectral variations caused by differences in sample thickness, particle size, or instrumental inconsistencies. By aligning all spectra to a common reference, MSC enhances comparability across samples and improves the accuracy of chemometric models [39].

By using the “**SpectralPreprocessingCupuassu.m”** MATLAB code, the baseline-corrected, SNV-corrected and MSC-corrected infrared spectra were obtained.

**BaselineSpectra_Cupuassu.xlsx:** This Excel file contains the preprocessed infrared spectral for dried cupuassu samples, where the raw spectra have been baseline-corrected using the Baseline correction method (**“BaselineCorrection.m”**). The structure of the dataset is as follows: the first column represents the wavenumbers (cm⁻¹) across the full infrared spectrum. Columns 2 to 28 contain the preprocessed spectral data for 27 cupuassu samples collected from three different growing zones. These zones are organized into three groups of columns: Zone 1 is represented by columns 2 to 10, containing nine baseline-corrected spectra from the first growing zone; Zone 2 is represented by columns 11 to 19, containing nine baseline-corrected spectra from the second growing zone; and Zone 3 is represented by columns 20 to 28, containing nine baseline-corrected spectra from the third growing zone. Each zone corresponds to a specific region of the spectrum, allowing for a detailed analysis of the spectral features across different samples and growing areas, all adjusted for baseline variations.

**SNVSpectra_Cupuassu.xlsx:** This Excel file contains the infrared spectral data for dried cupuassu samples, where the spectra (**“RawSpectra_Cupuassu.xlsx**” or “**BaselineSpectra_Cupuassu.xlsx”**) have been preprocessed using SNV correction (**“SNVSpectral.m”**). SNV could be applied to raw spectra or as part of a sequential preprocessing strategy (sequential application on raw to baseline and SNV) to assess the impact of each approach on various objectives, such as prediction, classification, and/or exploration. The structure of the dataset is as follows: the first column represents the wavenumbers (cm⁻¹) across the full infrared spectrum. Columns 2 to 28 contain the SNV-transformed spectral data for 27 cupuassu samples collected from three different growing zones. These zones are organized into three groups of columns: Zone 1 is represented by columns 2 to 10, containing nine SNV-transformed spectra from the first growing zone; Zone 2 is represented by columns 11 to 19, containing nine SNV-transformed spectra from the second growing zone; and Zone 3 is represented by columns 20 to 28, containing nine SNV-transformed spectra from the third growing zone. Each zone corresponds to a specific region of the spectrum, enabling a detailed analysis of the spectral features across different samples and growing areas after normalization.

**MCSSpectra_Cupuassu.xlsx:** This Excel file contains the infrared spectral data for dried cupuassu samples, where the spectra (**“RawSpectra_Cupuassu.xlsx**”, “**BaselineSpectra_Cupuassu.xlsx”** or “**SNVSpectra_Cupuassu.xlsx”**) have been preprocessed using MSC. MSC could be applied to raw spectra or as part of a sequential preprocessing strategy (sequential application on raw to baseline, SNV and MSC) to assess the impact of each approach on various objectives, such as prediction, classification, and/or exploration. The structure of the dataset is as follows: the first column represents the wavenumbers (cm⁻¹) across the full infrared spectrum. Columns 2 to 28 contain the MSC-corrected spectral data for 27 cupuassu samples collected from three different growing zones. These zones are organized into three groups of columns: Zone 1 is represented by columns 2 to 10, containing nine MSC-corrected spectra from the first growing zone; Zone 2 is represented by columns 11 to 19, containing nine MSC-corrected spectra from the second growing zone; and Zone 3 is represented by columns 20 to 28, containing nine MSC-corrected spectra from the third growing zone. Each zone corresponds to a specific region of the spectrum, facilitating the analysis of spectral features across different samples and growing areas, after correcting for scatter effects.

**Fig. 3:**
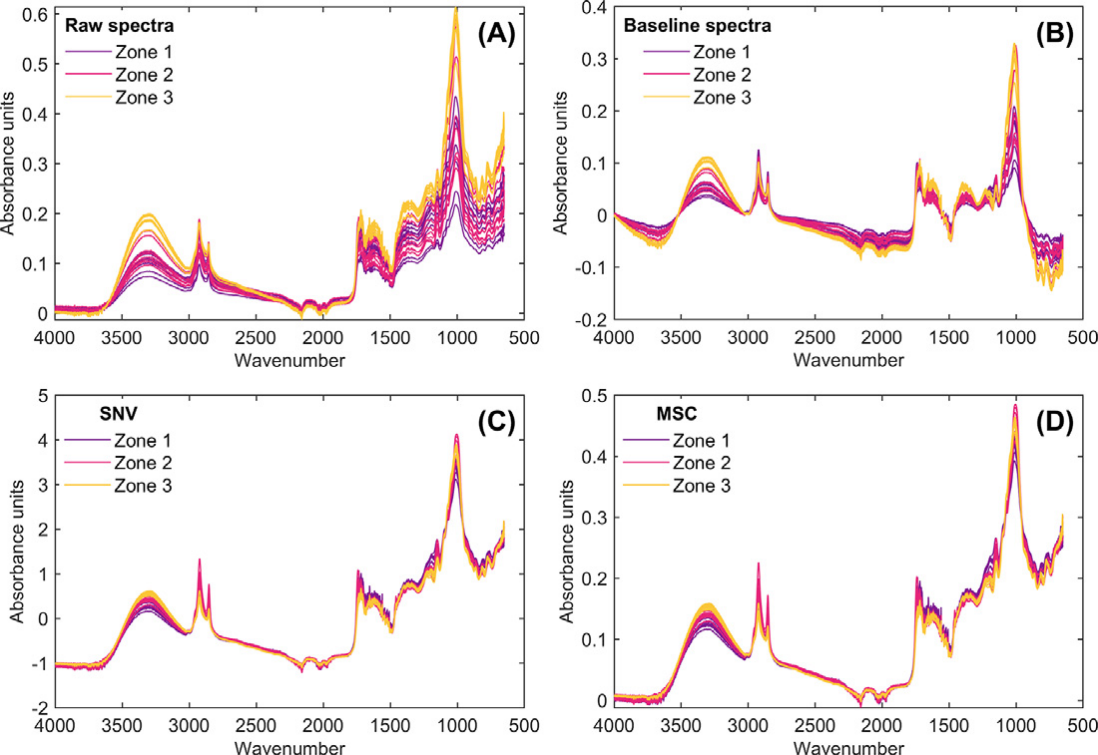
Infrared spectra and preprocessed spectral data of cupuassu pulp obtained by independently applying preprocessing techniques to raw infrared data. Raw spectra (A), baseline-corrected spectra (B), standard normal variate (SNV; C), and multiplicative scatter correction (MSC; D).

**Fig. 4:**
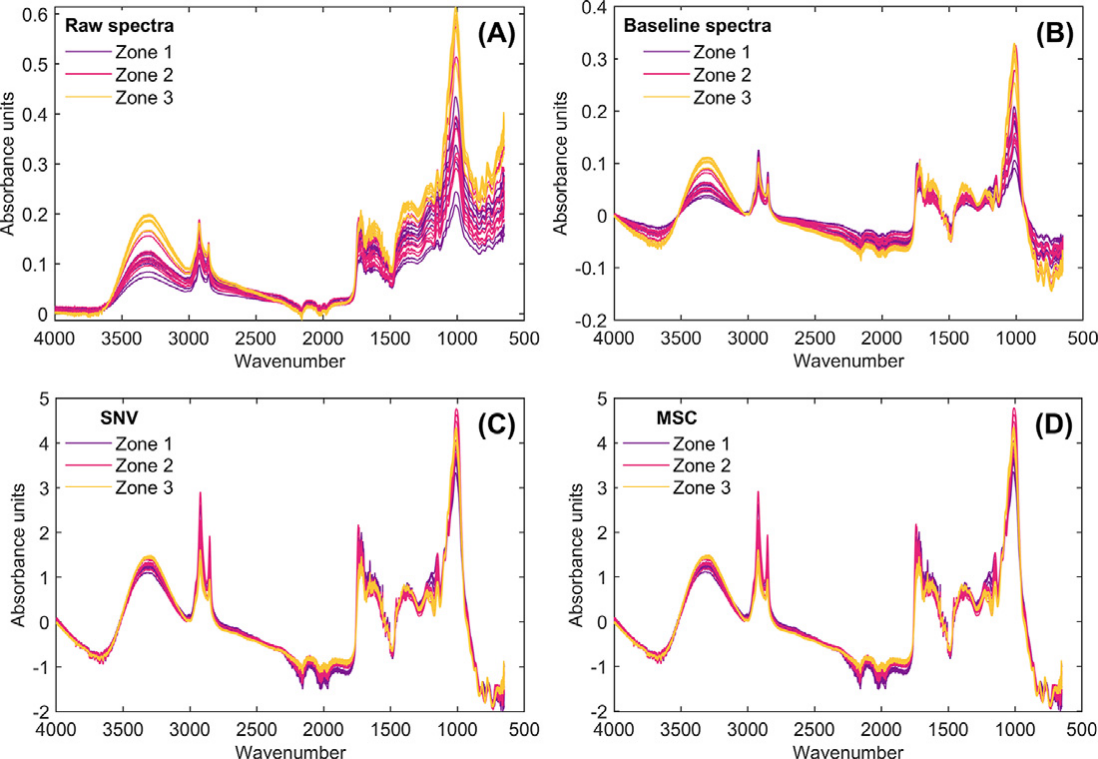
Infrared spectra and preprocessed spectral data of cupuassu pulp obtained by sequentially applying preprocessing techniques to raw infrared data. Raw spectra (A), baseline-corrected spectra (B), standard normal variate (SNV; C), and multiplicative scatter correction (MSC; D).

As a result of applying the **SpectralPreprocessingCupuassu.m** MATLAB script, the preprocessed infrared spectra are presented in Fig. 3, Fig. 4, corresponding to the independent and sequential preprocessing of the raw infrared dataset, respectively. As can be seen in both cases, differences arise in the spectra depending on whether preprocessing is applied independently or sequentially. These tools are useful for researchers to explore how different preprocessing approaches influence the outcomes of multivariate statistical models, allowing them to determine the most suitable conditions for spectral analysis.

**Fig. 5:**
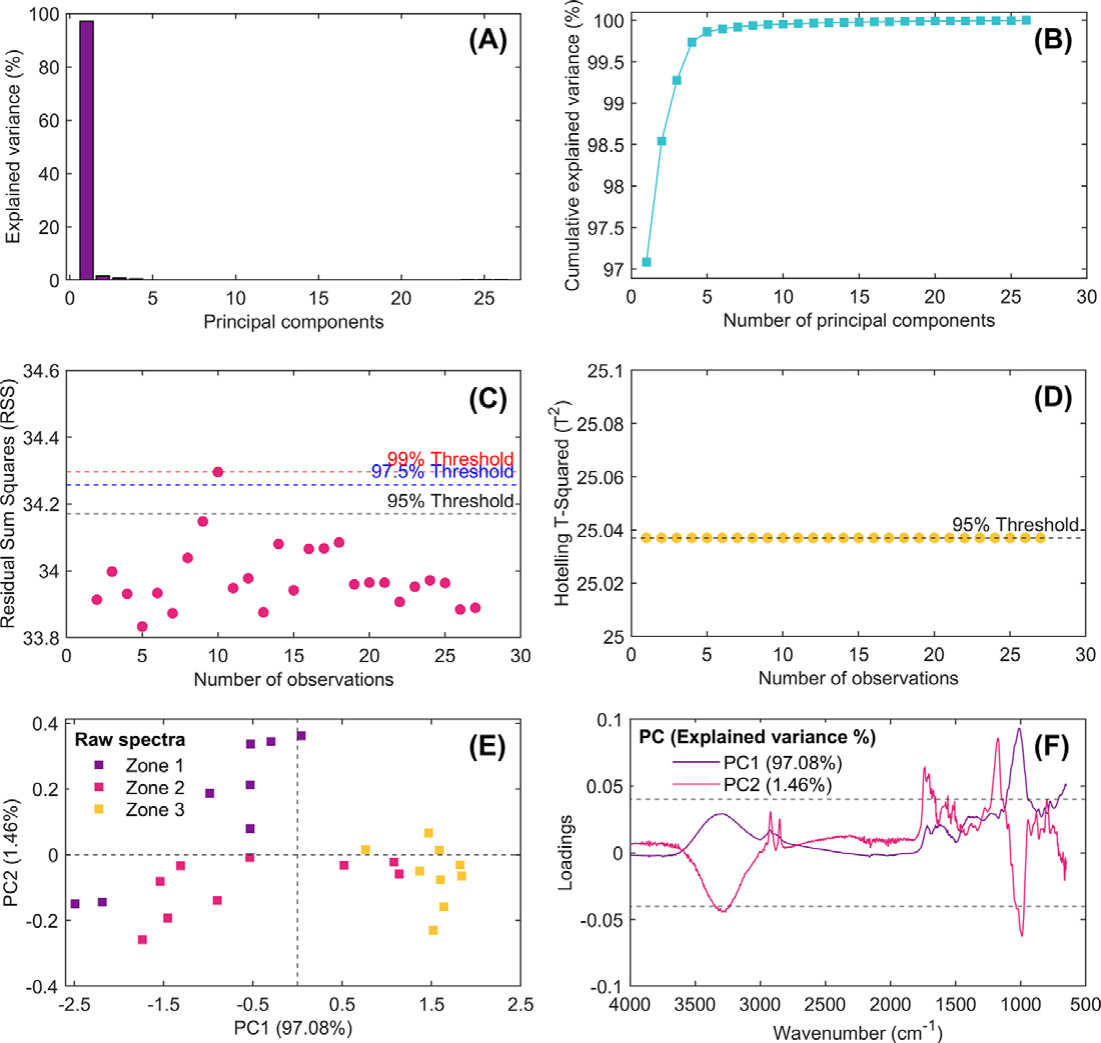
Results of multivariate statistical latent-based principal component model (PCA) obtained from exploratory analysis of raw spectra. Explained variance of computed principal components (A), cumulative explained variance vs. number of principal components (B), outlier detection using the residual sum of squares multivariate control chart (RSS; C), extreme outlier detection using the Hotelling’s T-Square multivariate control chart (T^2^; D), score plot of PC1 vs. PC2 (E), and loading spectra for PC1 and PC2 (F).

**Fig. 6:**
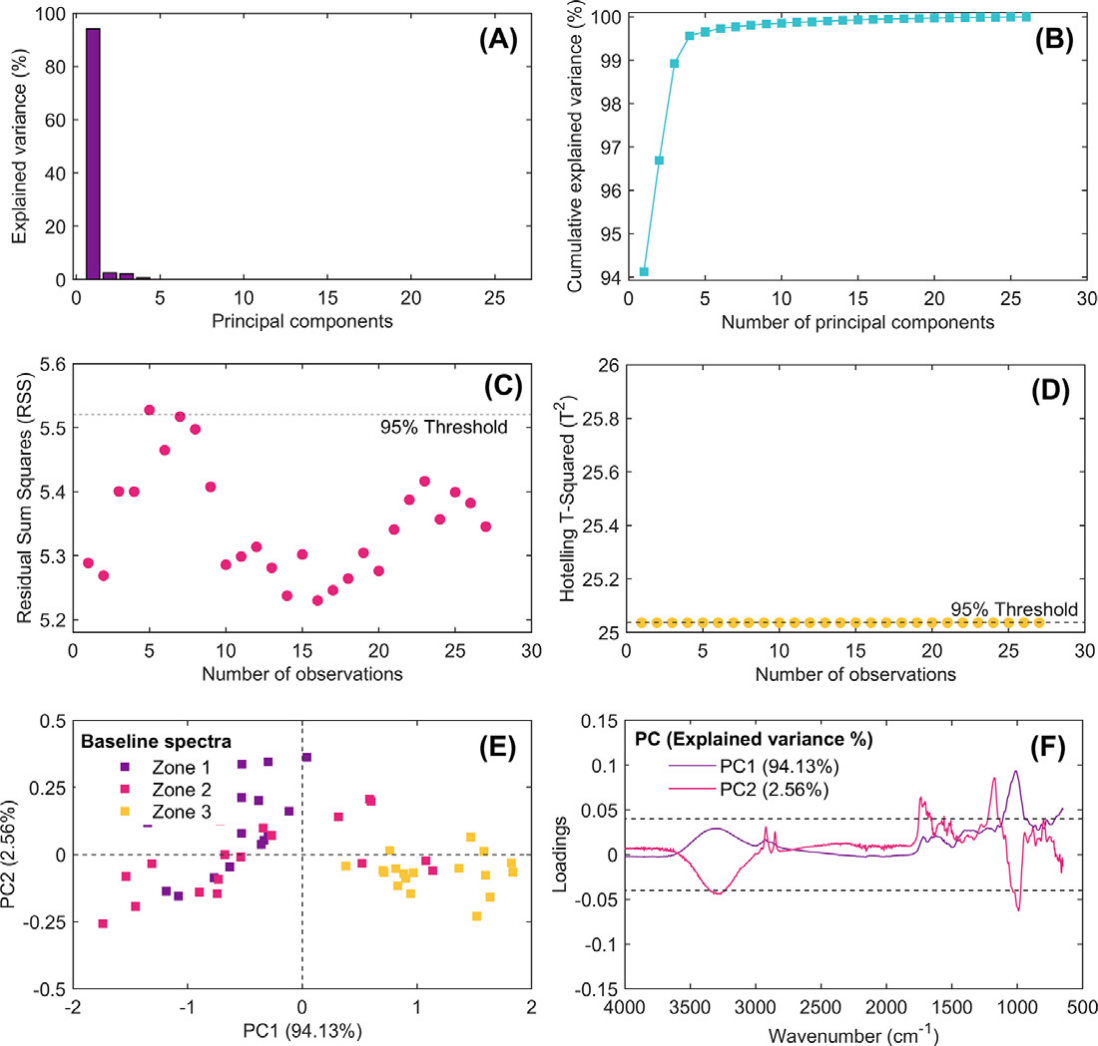
Results of multivariate statistical latent-based principal component model (PCA) obtained from exploratory analysis of baseline-corrected spectra. Explained variance of computed principal components (A), cumulative explained variance vs. number of principal components (B), outlier detection using the residual sum of squares multivariate control chart (RSS; C), extreme outlier detection using the Hotelling’s T-Square multivariate control chart (T^2^; D), score plot of PC1 vs. PC2 (E), and loading spectra for PC1 and PC2 (F).

**Fig. 7:**
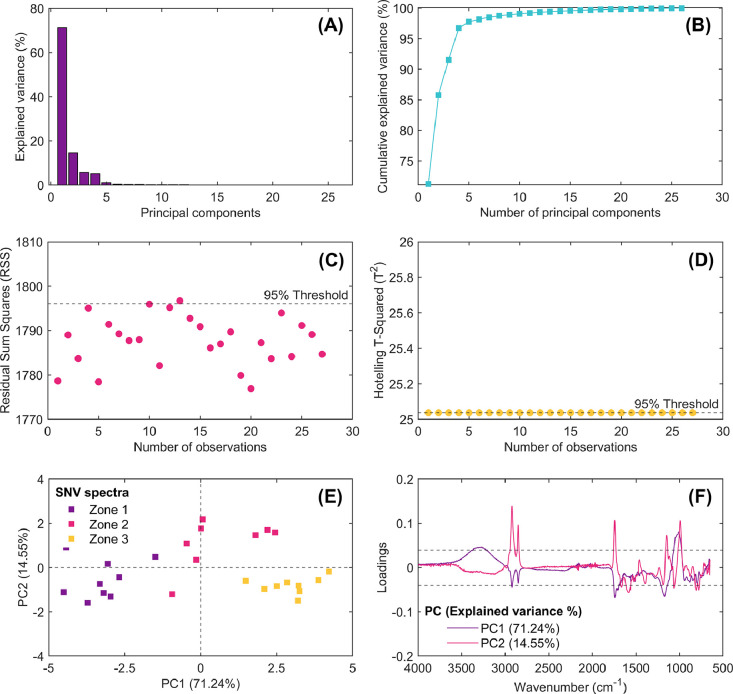
Results of multivariate statistical latent-based principal component model (PCA) obtained from exploratory analysis of standard normal variate (SNV) spectra. Explained variance of computed principal components (A), cumulative explained variance vs. number of principal components (B), outlier detection using the residual sum of squares multivariate control chart (RSS; C), extreme outlier detection using the Hotelling’s T-Square multivariate control chart (T^2^; D), score plot of PC1 vs. PC2 (E), and loading spectra for PC1 and PC2 (F).

**Fig. 8:**
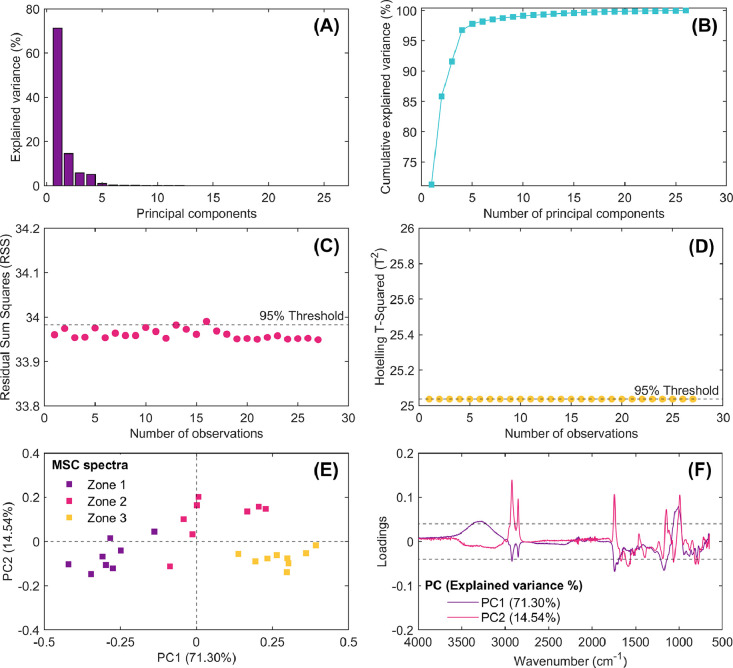
Results of multivariate statistical latent-based principal component model (PCA) obtained from exploratory analysis of multiplicative scatter correction (MSC) spectra. Explained variance of computed principal components (A), cumulative explained variance vs. number of principal components (B), outlier detection using the residual sum of squares multivariate control chart (RSS; C), extreme outlier detection using the Hotelling’s T-Square multivariate control chart (T^2^; D), score plot of PC1 vs. PC2 (E), and loading spectra for PC1 and PC2 (F).

**ExplorativePrincipalComponentAnalysisSpectral.m:** This MATLAB script was designed to apply PCA to a preprocessed spectral dataset. Using this code, the user is prompted to select one of four spectral preprocessing methods: raw spectra, baseline correction, SNV, or MSC, previously obtained in the abovementioned sections. Based on the user’s choice, the corresponding dataset is loaded, and its dimensions are determined. The maximum number of principal components that can be extracted is computed as the minimum of the number of spectra and wavenumbers minus one, and then, this maximal number of component are showed to users in MATLAB command window. The user is then required to specify the number of principal components for the analysis. PCA is performed using the Singular Value Decomposition (SVD) algorithm with mean-centering enabled. The principal component scores, loadings, explained variance, and residual statistics are computed. Several graphical representations are generated to aid in the interpretation of PCA results. These representations are summarized in Fig. 5, Fig. 6, Fig. 7, Fig. 8, including:

**i) Explained variance bar plot:** This bar plot visualizes the percentage of variance explained by each principal component (PC). Since PCA transforms the data into orthogonal components ordered by variance, this plot helps determine the importance of each PC (Figs. 5A, 6A, 7A and 8A). Higher variance means more information is retained. The first few PCs usually capture most of the dataset’s variability [40].
**ii) Cumulative explained variance plot:** This plot illustrates the cumulative sum of variance explained as more principal components are included (Figs. 5B, 6B, 7B and 8B). It is useful for deciding how many PCs should be retained to capture an adequate amount of information. The curve typically increases sharply at the beginning and then plateaus, indicating the point at which adding more PCs provides diminishing returns.
**iii) Residual sum of squares (RSS) plot:** The RSS plot measures the residual variance left after reconstructing the data using the selected principal components. Each observation’s residual error is computed, and statistical thresholds (95^th^, 97.5^th^, and 99^th^ percentiles) are marked (Figs. 5C, 6C, 7C and 8C). Points above these thresholds indicate potential outliers or observations that are not well represented by the selected PCs.
**iv) Hotelling’s T-Squared plot:** Hotelling’s T² statistic is used to assess the significance of each observation within the principal component space. This plot identifies samples that deviate significantly from the central distribution. Like the RSS plot, percentile thresholds (95^th^, 97.5^th^, and 99^th^) are included to highlight potential anomalies (Figs. 5D, 6D, 7D and 8D).
**v) Score plot:** This scatter plot represents the samples projected onto the first two principal components, PC1 and PC2. By reducing the dimensionality, patterns and clusters could be identified within the data. Different markers/colors are used to distinguish cupuassu growing areas or categories, making it easier to visualize relationships or separations in the spectral dataset (Figs. 5E, 6E, 7E and 8E).
**vi) Loadings plot:** This plot shows the contribution (weights) of each spectral variable (fingerprint) to the first two principal components (Figs. 5F, 6F, 7F and 8F). The loadings indicate how strongly each original variable influences the PCs. High absolute values suggest significant influence, while near-zero values indicate minimal contribution. This plot is crucial for interpreting which spectral features drive the observed variance.

Each of these figures plays a critical role in understanding the data structure, identifying patterns, detecting anomalies/outliers, and deciding how many principal components should be extracted from spectral dataset for further analysis. This script enables researchers to explore the impact of different preprocessing techniques on the spectral data and evaluate the computed principal components’ interpretability.

Future research should incorporate a broader range of samples originating from diverse geographical regions and climatic conditions, considering that cupuassu is cultivated throughout the Amazon basin and exhibits significant variability depending on its provenance. With regard to water desorption isotherms, further studies are needed to elucidate the effect of temperature on desorption behavior, as well as to quantify energy requirements and additional thermodynamic parameters that would enhance the understanding of cupuassu pulp properties and inform strategies for the revalorization of this by-product. Simultaneously, investigations into water adsorption isotherms, along with model-based approaches, should be undertaken to analyze the hysteresis phenomena between adsorption and desorption processes, given that the present work represents an initial step toward quantifying water desorption properties in cupuassu pulp. Although several studies have focused on cupuassu seeds, the pulp remains underexplored despite its significant potential as a raw material for food processing applications. Furthermore, future research should apply a variety of modeling strategies (using theoretical, empirical, semi-empirical and machine learning) to better characterize the isotherms across different temperatures and to establish robust computational tools for deriving critical thermodynamic parameters, such as differential and integral enthalpy-entropy relationships, thereby optimizing the storage, drying, and overall management of this valuable by-product.

## 4 Experimental Design, Materials and Methods

**Fig. 9:**
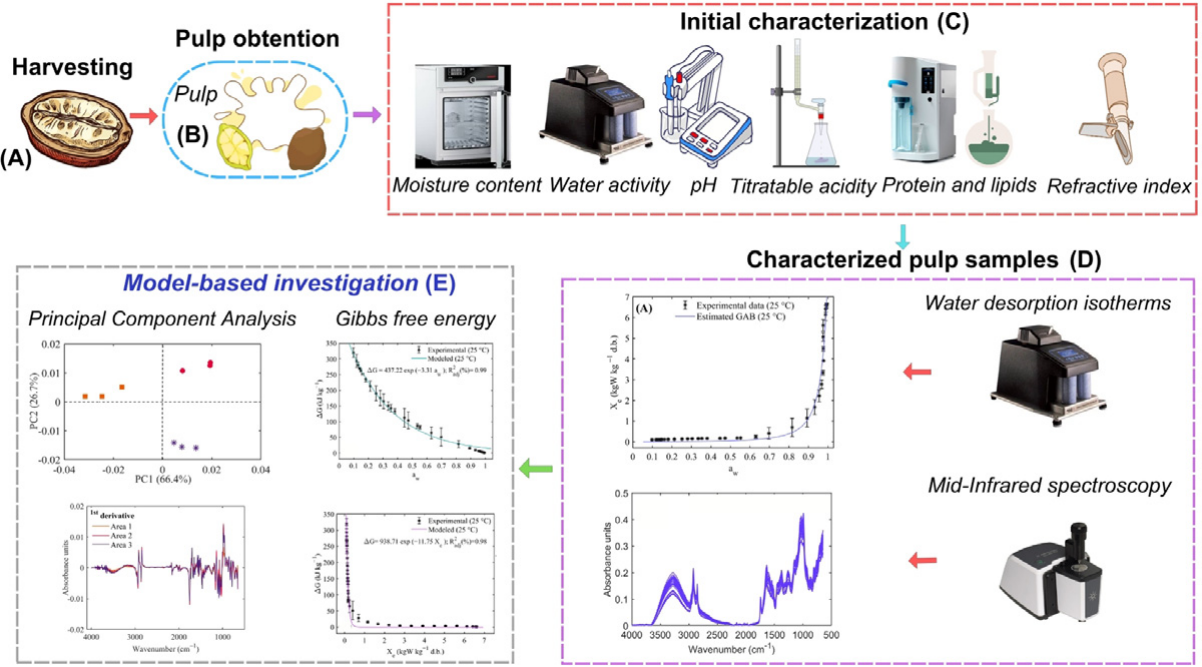
Experimental procedure followed for processing, characterization, dataset acquisition, and statistical analysis of water desorption and infrared spectral properties of cupuassu pulp.

**Fig. 10:**
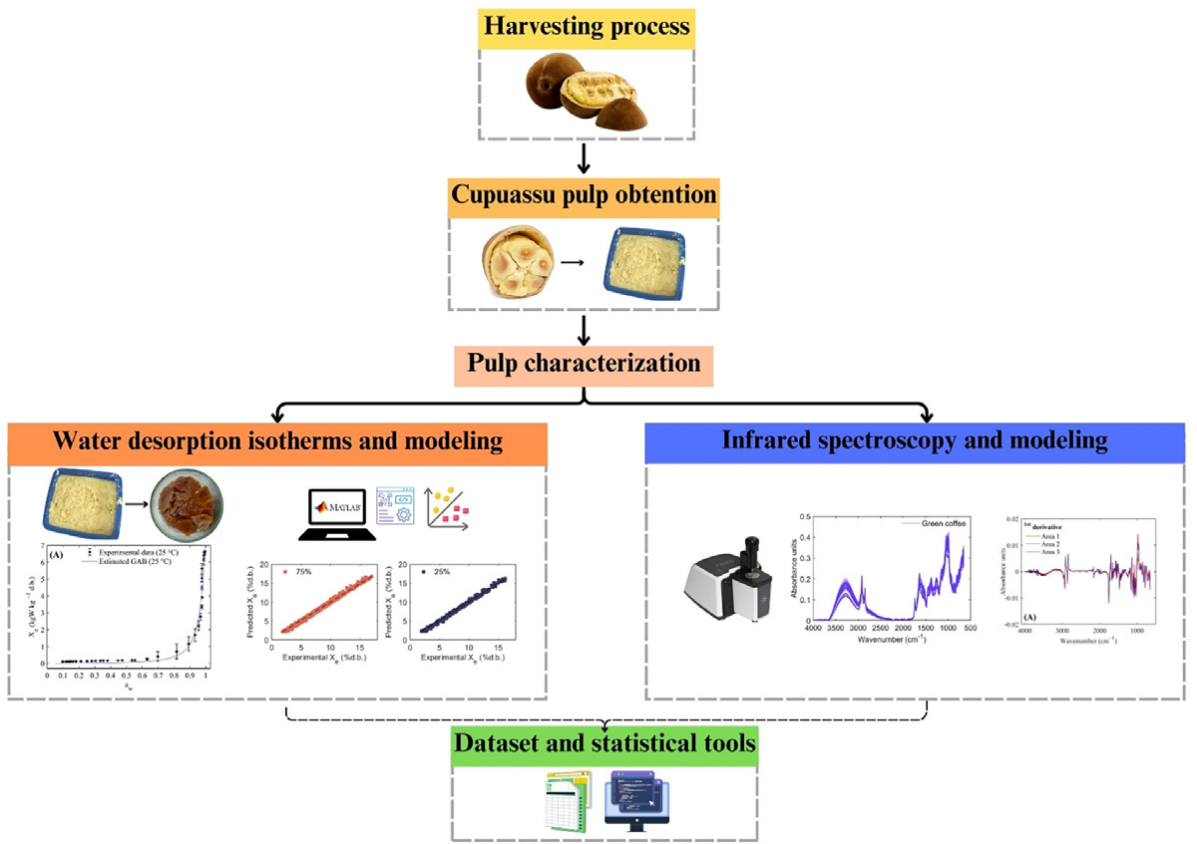
Flowchart illustrating the experimental procedure used to process cupuassu pulp samples and to obtain the dataset for multivariate statistical computer tools.

Fresh cupuassu fruits (*Theobroma grandiflorum* L., Fig. 9A) were obtained from various farmers three different growing areas in the Caquetá region of Colombia. Sample processing was carried out at the Amazonian Research Center CIMAZ-Macagual, Universidad de la Amazonia, located at an altitude of 250 meters above sea level (MASL), approximately 22 km south of Florencia, Caquetá, at coordinates 1° 37′ 00′' N latitude and 75° 36’ 00’’ W longitude. The cupuassu pulp was obtained by manually (Fig. 9B) separating it from the skin and seeds, followed by initial physicochemical characterization (Initial characterization section). These properties were moisture content, a_w_, pH, titratable acidity, protein, lipids and refractive index (Fig. 9C). The flowchart illustrating the experimental procedure followed to obtain the dataset and the multivariate statistical tools for the mathematical modeling of water desorption isotherms and infrared spectral properties of cupuassu pulp is shown in Fig. 10.

To determine moisture content, 10 g of cupuassu pulp samples were dehydrated in an oven (UF55, Memmert GmbH + Co.KG, Schwabach, Germany) at 105 °C for 24 hours, allowing the calculation of dry matter and moisture content. The a_w_ was assessed using 4 to 4.5 g of cupuassu pulp placed in a vapor sorption analyzer (VSA Aqualab Decagon Devices, Inc., Pullman, WA, USA). The dewpoint sensor was calibrated using four saturated aqueous salt solutions supplied by the instrument manufacturer: 13.41 m LiCl (0.25 ± 0.003 a_w_), 8.57 m LiCl (0.50 ± 0.003 a_w_), 6.0 m NaCl (0.76 ± 0.003 a_w_), and 2.33 m NaCl (0.92 ± 0.003 a_w_) [41]. For titratable acidity determination, 1 g of cupuassu pulp was diluted in 100 mL of distilled water, followed by the addition of 0.1 % phenolphthalein as an indicator. Titration was conducted using 0.1 N sodium hydroxide (NaOH) until reaching a pH of 8.2, and acidity was expressed as a percentage of citric acid [11]. pH was measured by mixing 5 g of pulp with 95 mL of water at 25 °C. Prior to analysis, the potentiometer (BP-3001, Trans Instruments, Singapore) was calibrated using buffer solutions at pH 4, 7, and 10 at 25 °C [42]. Further, the refractive index measurements were conducted using a digital refractometer (MA884, Milwaukee, USA).

To determine the lipid content, the Soxhlet extraction method was used. To achieve this, 2 g of pulp sample was refluxed in 50 mL of petroleum ether for 60 min, followed by an additional 30-min reflux in round-bottom flasks. Solvent removal was performed using a rotary evaporator at 45 °C until complete evaporation, and lipid content was expressed as a percentage. Crude protein was quantified using the Kjeldahl method, where 1 g of sample was digested with 15 mL of sulfuric acid and three Kjeldahl tablets in a digestion unit (Bloc-digest 12, JP Selecta, Spain). The digestion protocol included preheating at 400 °C for 30 min, followed by complete digestion at 420 °C for 1 h, controlled via an electronic regulator (RAT-2, JP Selecta, Spain). Distillation was carried out using 3% boric acid and 32 % NaOH in a Kjeldahl distiller (Pro-Nitro M, JP Selecta, Spain), with titration performed using a T-Shiro indicator and 0.1 N hydrochloric acid [43]. Crude protein content was calculated as nitrogen × 6.25.

The water desorption isotherms of cupuassu pulp samples were experimentally determined in triplicate using the Dynamic Dewpoint Isotherm (DDI) method, as provided by the vapor sorption analyzer (VSA, Aqualab Decagon Devices, Inc., Pullman, WA, USA; Fig. 9D). Prior to each experimental run, the VSA equipment was carefully calibrated according to the manufacturer’s specifications to ensure the accuracy and repeatability of the measurements. Calibration procedures included verification of dewpoint sensors and airflow rates to minimize any potential drift during the tests. The dewpoint sensor was calibrated using four saturated aqueous salt solutions supplied by the instrument manufacturer: 13.41 m LiCl (0.25 ± 0.003 a_w_), 8.57 m LiCl (0.50 ± 0.003 a_w_), 6.0 m NaCl (0.76 ± 0.003 a_w_), and 2.33 m NaCl (0.92 ± 0.003 a_w_). Approximately 5 g of pulp sample were placed inside the VSA sample chamber. The measurements were conducted at a controlled temperature of 25 °C using a constant dry airflow rate of 100 mL min^–1^. The a_w_ range for desorption was established from the initial aw of the fresh pulp (approximately 0.999) down to 0.1 a_w_, with incremental decreases of 0.01 a_w_ (Fig. 10). This controlled desorption process simulated conditions of gradual moisture loss that the pulp might undergo during drying. By following a systematic desorption protocol and maintaining uniform experimental conditions, the resulting water desorption isotherms reflected the thermodynamic equilibrium states between moisture content and water activity. This information is critical for modeling moisture-dependent phenomena such as drying kinetics, stability during storage, and the design of optimized dehydration or packaging processes. Subsequently, the water desorption isotherms were mathematically modeled using different sorption equations and the Gibbs free energy analysis was also achieved using the desorption isotherms (*Water desorption dataset and mathematical modeling* section; Fig. 9E).

The mid-infrared spectra of cupuassu (Fig. 9D), samples were acquired using an ATR-FTIR spectrometer (Cary 630, Agilent Technologies, Santa Clara, CA, USA) equipped with a horizontal diamond ATR accessory. Dehydrated cupuassu pulp samples (10 g) were first finely ground using a standard mill (Bunn G3 HD-Coffee Mill, Springfield, IL. USA) and sieved through a #60 mesh (particle size ≤ 0.25 mm) to ensure sample homogeneity by using a vibratory sieve shaker (EFL-2000, Endecotts LTD, London, U.K) for 15 min. Approximately 1 g of the powdered sample was carefully placed onto the ATR crystal and uniformly pressed to achieve optimal contact between the sample and the crystal surface. Spectra were recorded at room temperature (20 ± 0.5 °C) under controlled humidity conditions. A background spectrum was acquired prior to each measurement using the clean ATR crystal without sample to correct for environmental interferences.

Infrared spectra were collected in the range of 4000–650 cm^–1^, using a spectral resolution of 8 cm⁻¹ and averaging 20 scans per sample to enhance the signal-to-noise ratio. Each sample was analyzed in triplicate to ensure reproducibility of the measurements. To minimize systematic errors and enhance the quality of the spectral data, the raw spectra were subjected to baseline correction and normalization procedures. The baseline correction and advanced preprocessing techniques including SNV and MSC were done to enhance the quality of spectral information. Furthermore, latent-based chemometric model such as the PCA modeling (Fig. 9E) was conducted on the preprocessed data, as explorative tool to spectrally differentiate the cupuassu pulp obtained from different growing areas *(Infrared spectral data, preprocessing and latent-based mathematical modeling* section).

## Limitations

Not applicable.

## Ethics Statement

The dataset acquired in this study did not involve human subjects, animal experiments, or data obtained from social media platforms.

## CRediT Author Statement

**Andrés F. Bahamon-Monje:** Conceptualization, Data curation, Formal analysis, Software, Investigation, Methodology, Writing–original draft, Writing–review and editing. **Paola A. García-Rincón:** Conceptualization, Data curation, Formal analysis, Investigation, Methodology, Writing–original draft, Writing–review and editing. **Gentil. A. Collazos-Escobar:** Data curation, Formal analysis, Investigation, Methodology, Software, Validation, Visualization, Writing–original draft, Writing–review and editing. **Claudia M. Amorocho-Cruz:** Conceptualization, Data curation Investigation Methodology, Writing–review and editing. **Nelson Gutiérrez-Guzmán:** Conceptualization, Project administration, Resources, Supervision, Validation, Writing–review and editing.

## Data Availability

Mendeley DataDataset and statistical tools for mathematical modeling of water desorption isotherms and infrared latent-spectral properties of cupuassu pulp (Original data)
